# Supplementary material for: Transgenic East African Highland Banana Plants Are Protected against Radopholus similis through Host-Delivered RNAi
Source: Int J Mol Sci. 2023 Jul 28;24(15):12126. doi: 10.3390/ijms241512126 (PMC10418933; doi:10.3390/ijms241512126)
Supplement: Supplementary file 1 [file ijms-24-12126-s001.zip › Figure S1 - Multiplex PCR to detect expression levels of target genes after dsRNA soaking.pptx]

## Slide 1
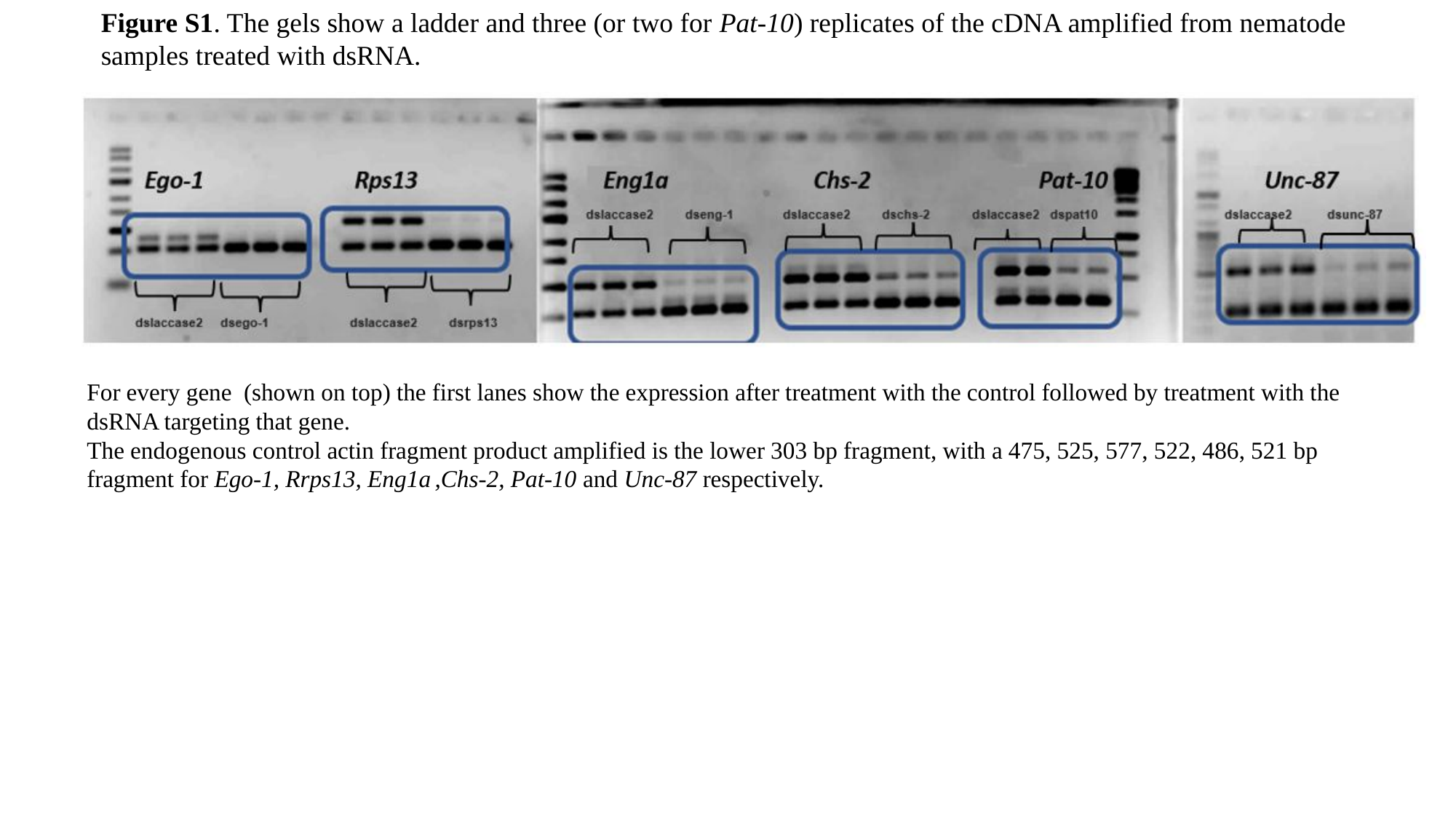

Figure S1. The gels show a ladder and three (or two for Pat-10) replicates of the cDNA amplified from nematode samples treated with dsRNA.
For every gene (shown on top) the first lanes show the expression after treatment with the control followed by treatment with the dsRNA targeting that gene.
The endogenous control actin fragment product amplified is the lower 303 bp fragment, with a 475, 525, 577, 522, 486, 521 bp fragment for Ego-1, Rrps13, Eng1a ,Chs-2, Pat-10 and Unc-87 respectively.
