# Supplementary material for: Transgenic East African Highland Banana Plants Are Protected against Radopholus similis through Host-Delivered RNAi
Source: Int J Mol Sci. 2023 Jul 28;24(15):12126. doi: 10.3390/ijms241512126 (PMC10418933; doi:10.3390/ijms241512126)
Supplement: Supplementary file 1 [file ijms-24-12126-s001.zip › Figure S2-nematode target sequences fasta.pdf]

Figure S2- Nematode Target Sequences

>CHITIN SYNTHASE (477bp)
GGCCGGGGCTTCTCGATGGAGTCCAATGTGCAGCTGATGTACGCCAAAATAGCGTCGGTC
ATCTACGCATTCATCATGCTGGCCGTGCTGATCGCTACCACCAATCAAATCGTGTTGGAG
ACGGTGTTCTCGCCCACGTCGATGTTCGTGCTGGGAATGGTGTGCATCTTCTCCTTCGCC
TCCTGCATCCACCCCAAAGAGTTCTCCAACATCGTCTTCGGCACGGTCTTCTTTCTGATG
ATCCCCTCCACGTACGTGTTCCTCTCCCTCTACTCCCTGATCAATCTGAACGTGATCAAC
TGGGGCACACGAGAGGCGGTTGCCAAAGCTACGGGACAAGTCTCAGAGAGCCTTGCGGGG
CGCATGTTGCGCCGAGTGATCAACACCAGCGACGACAGTTCGGCCTTGGCGCGCTTGCTC
GGTCGATTTCGCACCAGCGAAGAGTCCAACGAAAAAATCGTCACATTGGAGAAGAAG
>EGO-1 (312bp)
AAATCCGTCAATCGTTGCCGGCGACATTCGCATGTTCGAAGCTGTTGACATTCCCGCACT
GCACCACCTCTGCGACGTGGTCGTCTTCCCGCGCTACGGACCGCGTCCGCACACTGATGA
GATGGCCGGCTCCGATCTGGACGGGGACGAGTACACGGTGATTTGGGACGAGCAGCTGTA
CCTGGACCGGAATGAGGAGGCATTCGATTACACCTCGAAGTGGGCAAAGGGTGCAATATT
TTATTTAATATGGTCATTTTTTGTTTAGAGCAACAGAGGCACCAGCCATAGACGAAAAGG
ACCTGGTATGCA
>BETA 1,4 ENDOGLUCANASE (500bp)
ATGAACTGCTTGTTCCTTTTGCCTCTGTTCTTCGCCTTGGCCGGCGCCGCCGATCCCCCC
TACGGAGCGCTGTCCGTCTCCGGGACCAATCTGAAGGGCTCGGGCGGCCAGAATGTGGCG
CTGCACGGGATGTCGCTGTTCTGGTCGCAGTGGGAGTCCGAGTTCTACAACGAGGAGACA
GTGCGTGCGCTCAAATGCCAGTGGAACTCGAACGTGGTGCGCGCCGCCATGGCTGTGGAG
GAGGGCGGCTACCTGAGCAACCCATCGGCGGAGCAGGCACGTGTCGAGGCGGTGATCAAC
GCAGCCATCAGCCAGGGCATCTACGTTATCGTGGACTGGCACGACCACAACGCGCAGAAC
CACGGGGACCAGGCAGTGGCCTTCTTCACAGCGATCGCCCAGAAGTACGGCTCCAATCCG
CATGTGCTGTACGAGATCTTCAACGAGCCCCTCCAAGTGGACTGGAGCAGCGTGATCAAG
CCATACGCCGAGAGAGTGAT
>PAT-10 (420bp)
GTTCGACCGCGGAAAGAACGGCTACATCATGGCCACTCAGATCGGAGTGATCATGAACGC
CATGGAGCAAGACTTCGACGAGAAGACGCTGCGGAAGTGAGCAAATCCGCCAAATTGCAA
TTGATTGATTTCATTTCAGACTGATCCGCAAGTTTGACGCTGACGGCTCCGGCAAGCTGG
AGTTCGACGAGTTCTGCGCTCTGGTGTACACTGTGGCCAACACGGTGGACAAGGACACGT
TGCGCAAGGAGCTGAGGGAGGCGTTCCGCCTGTTCGATAAGGAGGCAAGCCAAACAGAAA
ACAACCAGTTGATTGACGTGTTCCATCCGTTCAGGGCAACGGCTACATCTCTCGCCCCAC
GCTGAAGGGCCTGCTGAAAGAGATCGCGCCCGACCTCAGCGACAAGGACTTGGACGCAGC
G
>RPS13 (388bp)
CCAGCAAATTAGCCGACAACAACTGCAATCATGGGTCGTATGCACAATCCTGGAAAGGGA
ATATCGCGTTCTGCGCTCCCATATCGGCGATCAGTCCCCACATGGCAAAAGTTGACCAAC
CAAGATGTGGAGGAACAGATCGTGAAGTTGGCTCGAAAAGGACTGCGCCCATCCCAGATT
GGTGTCATTCTTCGTGACTCTCATGGAGTGGCACAGGTCCGCCGCATTACAGGGAACAAG
ATCGTCCGCATTCTCCGATCAAAAGTGATTTAAAATATCACTGATTGCGATTAATAGAAA
TGTTACAATTTAGGGAATGGCTCCCGAAATTCCGGAAGATCTTTACCATCTTATCAAGAA
GGCAGTGAGCATCCGCAAACACCTGAAG
>UNC-87 (393bp)
GTACAACCACGAGCAGTCCATCGACCAGACCAGCATTCCCTACCAGATGGGGTCGAACAA
GTATGCATCCCAGAAGGGCATGACCGGGTTCGGACAGCCGCGCTGGGAGGTGCTGGACCC
GTCGATCAGCTACCAGAACCGCAAGTCGCAAGGTTTGCAGAGAATCTGGCGTTCAATTCT
AGTTTTGATATTGCCCAGGAATGGTCCGCCTCCAGTCCGGCACCAACCGGTTCGCGTCCC
AGGCTGGCATGACTGGCTTCGGGACGCCGAGGAACACCACCTATGAGGCGGAGGCGGGTG
AACTGCCCTACGACGACATGAAGAAGTCGGAGGCGATCATCCCGTCCCAAGCCGGATGGA
ACAAGGGAGACTCGCAAAAGGTACGGCAGAACA
