# Supplementary material for: Transgenic East African Highland Banana Plants Are Protected against Radopholus similis through Host-Delivered RNAi
Source: Int J Mol Sci. 2023 Jul 28;24(15):12126. doi: 10.3390/ijms241512126 (PMC10418933; doi:10.3390/ijms241512126)
Supplement: Supplementary file 1 [file ijms-24-12126-s001.zip › Table S2-Regression analysis to relate necrosis and plant height.pdf]

## Analysis of Variance

A

| Source | DF | Adj SS | Adj MS | F-Value | P-Value |
| --- | --- | --- | --- | --- | --- |
| Regression | 7 | 129880 | 18554 | 605.48 | 0.000 |
| NI | 1 | 115527 | 115527 | 3770.00 | 0.000 |
| Test plant group | 6 | 5073 | 846 | 27.59 | 0.000 |
| Error | 622 | 19060 | 31 | | |
| Lack-of-Fit | 402 | 12741 | 32 | 1.10 | 0.208 |
| Pure Error | 220 | 6319 | 29 | | |
| Total | 629 | 148941 | | | |

## Coefficients

| Term | Coef | SE Coef | T-Value | P-Value | VIF |
| --- | --- | --- | --- | --- | --- |
| Constant | 104.197 | 0.667 | 156.17 | 0.000 | |
| NI | -0.6141 | 0.0100 | -61.40 | 0.000 | 1.30 |
| Test plant group | | | | | |
| dsCS-Laccase-2 | 9.795 | 0.875 | 11.19 | 0.000 | 1.93 |
| dsEng1a | 6.274 | 0.833 | 7.53 | 0.000 | 1.75 |
| dsPat-10 | 5.634 | 0.826 | 6.82 | 0.000 | 1.72 |
| dsRps13 | 3.538 | 0.825 | 4.29 | 0.000 | 1.71 |
| dsUnc-87 | 4.865 | 0.831 | 5.86 | 0.000 | 1.74 |
| non-transformed | 9.476 | 0.880 | 10.77 | 0.000 | 1.95 |

## Model Summary

B

| S | R-sq | R-sq(adj) | R-sq(pred) |
| --- | --- | --- | --- |
| 5.53569 | 87.20% | 87.06% | 86.88% |

## Regression Equation

C

| Test plant group | |
| --- | --- |
| dsChs-2 | plant height = 104.197 - 0.6141 NI |
| dsCS-Laccase-2 | plant height = 113.991 - 0.6141 NI |
| dsEng1a | plant height = 110.471 - 0.6141 NI |
| dsPat-10 | plant height = 109.830 - 0.6141 NI |
| dsRps13 | plant height = 107.735 - 0.6141 NI |
| dsUnc-87 | plant height = 109.061 - 0.6141 NI |
| non-transformed | plant height = 113.672 - 0.6141 NI |
