# Supplementary material for: Transgenic East African Highland Banana Plants Are Protected against Radopholus similis through Host-Delivered RNAi
Source: Int J Mol Sci. 2023 Jul 28;24(15):12126. doi: 10.3390/ijms241512126 (PMC10418933; doi:10.3390/ijms241512126)
Supplement: Supplementary file 1 [file ijms-24-12126-s001.zip › Table S3- Primers used in this study .pdf]

Table S3: Primers used in this study

FORWARD PRIMER	TARGET	FORWARD SEQUENCE	REVERSE PRIMER	REVERSE SEQUENCE	PURPOSE	TEMPLATE	TA	PRODUCT
**R.S-ACTIN_SQ_F**	actin	gacgaggcgcagtccaag	R.S-actin_SQ_R	gccagagtcgagcacaatg	RT-PCR	*R. similis cDNA*	57	303
**CHS-ASENSE-BAMH1F**	Chs-2	ccggatccggccggggcttctcgatggagt	chs Asense-mlu1R	ggcacgcgtgttggactcttcgctggtg	cloning	pGemt cloned vector	54	443
**CHS-SENSE-NOTF**	Chs-2	gggcggccgcggccggggcttctcgatggagt	chs sense-xho1R	ggcctcgaggttggactcttcgctggtg	cloning	pGemt cloned vector	54	443
**SCHS-F**	Chs-2	gaacctgaagagcgacgttg	Schs-R	caagtccagtgtgttcgcaa	RT-PCR	*R. similis cDNA*	54	522
**6AF**	Chs-2	gttggactcttcgctggtg	UbiRev	tttagccctgccttcatacg	colony PCR	Bacterial colony	54	554
**CHSF**	Chs-2	ttctgatgatcccctccacg	IU-Rc	ggcatgcgaactctacttgg	detection	transgenic plant gDNA	56	659
**EGO1-SENSE F**	Ego-1	aaatccgtcaatcgttgccg	ego1 sense R	tgcataccaggtccttttcg	Isolation	*R. similis cDNA*	54	312
**EGO1-ASENSE-BAMH1F**	Ego-1	ccggatccaaatccgtcaatcgttgccg	ego1 Asense-mlu1R	ggcacgcgttgcataccaggtccttttcg	cloning	pGemt cloned vector	53	312
**EGO1-SENSE-NOTF**	Ego-1	gggcggccgcaaatccgtcaatcgttgccg	ego1 sense-xho1R	ggcctcgagtgcataccaggtccttttcg	cloning	pGemt cloned vector	55	313
**SEGO-F**	Ego-1	gggcaagcgatatgaagtgg	Sego-R	agtcagattcaaacccgtgat	RT-PCR	*R. similis cDNA*	54	475
**7AF**	Ego-1	cgagtgcataccaggtcctt	UbiRev	tttagccctgccttcatacg	colony PCR	Bacterial colony	55	420
**ENG1A-SENSE F**	Eng1a	atgaactgcttgttccttttgc	eng1a-sense R	atcactctctcggcgtatgg	Isolation	*R. similis cDNA*	55	500
**ENG1A-ASENSE**	Eng1a	ggggatccatgaactgcttgttccttttgc	eng1a-Asense mlu1-	ggcacgcgtatcactctctcggcgtatgg	cloning	pGemt cloned vector	55	500
**ENG1A-SENSE NOT1-F**	Eng1a	cagcggccgcatgaactgcttgttccttttgc	eng1a-sense xho1-R	ggctcgagatcactctctcggcgtatgg	cloning	pGemt cloned vector	55	501
**ENG1A-SENSE NOT1-F**	Eng1a	cagcggccgcatgaactgcttgttccttttgc	eng1a-sense xho1-R	ggctcgagatcactctctcggcgtatgg	cloning	pGemt cloned vector	55	501
**SENDO-F**	Eng1a	tcttggacggtctgagcatt	Sendo-R	aatctggttgcccgtgtttc	RT-PCR	*R. similis cDNA*	55	577
**8AF**	Eng1a	cgtatggcttgatcacgctg	UbiRev	tttagccctgccttcatacg	colony PCR	Bacterial colony	53	591
**ENG1AF**	Eng1a	tctccgggaccaatctgaag	IU-Re	agcaacacaatcgttcccag	detection	transgenic plant gDNA	56	563
**BMC_INTRON-F**	hairpin loop	cgcgtcctacctgtcaaaga	BMC_intron-R	tcatttctcttgcctgactgtg	colony PCR	Bacterial colony	54	550
**LOOPF**	hairpin loop	tctgcaggaaatggtagggt	LoopR	ggcatgcgaactctacttgg	detection	transgenic plant gDNA	56	228
**NPT11-F**	Kanamycin	agcaatatcacgggtagcca	nptII-R	ttgggtggagaggctattcg	detection	transgenic plant gDNA	55	646
**KANF**	Kanamycin	tattcggctatgactgggca	kanR	cctgatgctcttcgtccaga	detection	transgenic plant gDNA	56	435
**PAT-10-F**	Pat-10	gagatcctcgccgagattga	pat-10-R	ccattcctgtgtgttccgtc	Isolation	*R. similis cDNA*	53	506

**PAT10 SENSE F**	Pat-10	gttcgaccgcggaaagaacg	pat10 sense R	tcgggcgcgatctctttcag	cloning	pGemt cloned vector	56	506
**RS-PAT_ANTISENSE-F**	Pat-10	ccggatcctcgaggaataccagcgcttc	RS-PAT_antisense-R	ggacgcgttctttcagcaggcccttcag	cloning	pGemt cloned vector	52	316
**RS-PAT_SENSE-F**	Pat-10	ccgcggccgctcgaggaataccagcgcttc	RS-PAT_sense-R	ggctcgagtctttcagcaggcccttcag	cloning	pGemt cloned vector	53	316
**SPAT-F**	Pat-10	catggagcaagacttcgacg	Spat-R	atggccgcgtgtttattgtt	RT-PCR	*R. similis cDNA*	54	486
**9AF**	Pat-10	cccgacctcagcgacaag	UbiRev	tttagccctgccttcatacg	colony PCR	Bacterial colony	55	510
**PAT10F**	Pat-10	gaagtgagcaaatccgccaa	IU-Rp	acagttgtagtcgagcgtga	detection	transgenic plant gDNA	55	513
**RS-RPS13_ANTISENSE-F**	Rps13	ggggatccccagcaaattagccgacaaca	RS-rps13_antisense-R	ggcacgcgtcttcaggtgtttgcggatgc	cloning	pGemt cloned vector	60	405
**RS-RPS13_SENSE-F**	Rps13	gggcggccgcccagcaaattagccgacaaca	RS-rps13_sense-R	ggcctcgagcttcaggtgtttgcggatgc	cloning	pGemt cloned vector	60	407
**SRPS13-F**	Rps13	tgggtcgtatgcacaatcct	Srps13-R	tcgacgactcatacttccagg	RT-PCR	*R. similis cDNA*	54	525
**10AF**	Rps13	ctcgagcttcaggtgtttgc	UbiRev	tttagccctgccttcatacg	colony PCR	Bacterial colony	52	498
**RPS13F**	Rps13	caactgcaatcatgggtcgt	IU-Rr	agcaacacaatcgttcccag	detection	transgenic plant gDNA	56	508
**UNC-F**	Unc-87	cgctcaggtacgaacaagtt	unc-R	gcttgcccttcacgtcac	Isolation	*R. similis cDNA*	56	617
**RS_UNC_ANTISENSE- F**	Unc-87	ccggatccgtacaaccacgagcagtcca	RS_UnC_antisense- R	ggctcgaggttctgccgtaccttttgcg	Cloning	pGemt cloned vector	54	408
**RS_UNC_SENSE-F**	Unc-87	ccgcggccgcgtacaaccacgagcagtcca	RS_UnC_sense-R	ggctcgaggttctgccgtaccttttgcg	Cloning	pGemt cloned vector	55	410
**SUNC-F**	Unc-87	gttcctctactgtccaccgt	Sunc-R	ctcccaaatgcgcttcaagt	RT-PCR	*R. similis cDNA*	56	521
**11AF**	Unc-87	ttgggtggagaggctattcg	UbiRev	tttagccctgccttcatacg	colony PCR	Bacterial colony	54	501
**UNC87F**	Unc-87	gacatgaagaagtcggaggc	IU-R	cacccaggcaccgaatttac	detection	transgenic plant gDNA	55	643
