# Supplementary material for: Transgenic East African Highland Banana Plants Are Protected against Radopholus similis through Host-Delivered RNAi
Source: Int J Mol Sci. 2023 Jul 28;24(15):12126. doi: 10.3390/ijms241512126 (PMC10418933; doi:10.3390/ijms241512126)
Supplement: Supplementary file 1 [file ijms-24-12126-s001.zip › Table S4 - Composition of soaking solution comprising M9 buffer.pdf]

Table S4: Composition of soaking solution comprising M9 buffer, gelatin, spermidine and dsRNA

#	COMPONENT	STOCK	FINAL	REACTION VOLUME (µL)
1	M9 soaking Buffer	5x	1x	6
2	Gelatin	0.55%*	0.05%	2.72
3	Spermidine (Sigma S2626)	100mM*	3mM	0.9
4	dsRNA	10mg/ml	2mg/ml	6
5	Nematode suspension	-		14.38
	**Total volume**			**30**

*A 0.1M stock solution of spermidine was prepared by dissolving 0.145 g in 10 ml of water followed by filter-sterilizing through a 0.22 µm filter and storage at −20 °C for single use.

*A 0.55 % (w/v) solution of gelatin was prepared by adding 0.55 g of gelatin to a total volume of 100 ml of H_2O then autoclaved at 121 ºC for 15 minutes at 15 psi (1.05 kg/cm ^{2}).
